# Is High Maternal Body Mass Index Associated with Cesarean Section Delivery in Mongolia? A Prospective Observational Study

**DOI:** 10.31372/20190403.1056

**Published:** 2019

**Authors:** Naoko Hikita, Megumi Haruna, Masayo Matsuzaki, Emi Sasagawa, Minoru Murata, Ariunaa Yura, Otgontogoo Oidovsuren

**Affiliations:** a Department of Midwifery and Women’s Health, Division of Health Sciences and Nursing, Graduate School of Medicine, The University of Tokyo, Tokyo, Japan; b Department of Children and Women’s Health, Division of Health Science, Graduate School of Medicine, Osaka University, Japan; c Mito Saiseikai General Hospital, Ibaraki, Japan; d Darkhan-Uul General Hospital, Darkhan-Uul, Mongolia; e Pharmaceutical Department, Mongolian National University of Medical Sciences, Darkhan-Uul Medical College, Mongolia

**Keywords:** maternal obesity, maternal health, cesarean section, Mongolia

## Abstract

More than half of women are reported to be overweight or obese in Mongolia, thus becoming a big health issue. Though maternal obesity is a known risk factor for cesarean section (CS), it remains unclear how much a high maternal body mass index (BMI) would influence the risk of CS among pregnant women in Mongolia. This study aimed to investigate whether a higher maternal BMI is related to CS delivery in Darkhan-Uul Province, Mongolia. Pregnant women at 20 weeks’ gestation or less who visited public health facilities for antenatal health checkups between November 2015 and January 2016 were recruited. Data were collected using self-administered questionnaires, body weight measurement, and medical records. In total, 508 pregnant women participated, and data from 336 women were analyzed. Multiple logistic regression analysis showed that higher maternal BMI at first antenatal care visit (adjusted odds ratio [AOR] = 1.102, *p* = .033), higher gestational weight gain (AOR = 1.111, *p* = .001), older maternal age (AOR = 1.076, *p* = .030), and gestational age at delivery (AOR = 0.765, *p* = .005) were associated with CS delivery. This study is the first to demonstrate that higher maternal BMI and higher gestational weight gain are associated with CS delivery in Mongolia. Moreover, older maternal age and lower gestational age at delivery were found to be associated with CS delivery. Preventing obesity among women is important not only from the viewpoint of prevention of lifestyle diseases but also from the obstetric point of view; it is important for medical personnel to communicate the importance of preventing obesity to all women in Mongolia.

## Introduction

The prevalence of obesity, which has increased worldwide, is a public health concern (NCD Risk Factor Collaboration, 2017; World Health Organization, 2018) because obesity is a risk factor for several non-communicable diseases such as hypertension, coronary heart disease (Manson et al., 1990), stroke (Song, Sung, Davey Smith, & Ebrahim, 2004), and many cancers (Calle, Rodriguez, Walker-Thurmond, & Thun, 2003; Reeves et al., 2007). In Mongolia, 49.0% of 15–64-year-old men and 59.9% of 15–64-year-old women are either overweight (25.0 kg/m^2^ ≤ body mass index (BMI) < 30.0 kg/m^2^) or obese (BMI ≥ 30 kg/m^2^) (Public Health Institute of the Ministry of Health and Sports, 2013). Even among women aged 25–34 years, 46.2% are either overweight or obese; thus, obesity has become one of the major health issues in the country. Increased BMI among women is a serious concern because high BMI during pregnancy is associated with gestational diabetes mellitus, preeclampsia, venous thrombo-embolism, miscarriage, and cesarean section (CS) (Fitzsimons, Modder, & Greer, 2009).

CS is a mode of delivery effective in preventing maternal, neonatal, and infant mortality (Ye, Betrán, Guerrero, Souza, & Zhang, 2014). In 1985, the World Health Organization recommended the ideal CS rate to be between 10 and 15% (World Health Organization, 1985). However, the CS rates are increasing worldwide despite this recommendation (Ye et al., 2014; World Health Organization, 2015) because maternal age and the prevalence of obesity are increasing (Ye et al., 2014). In Mongolia, CS rates have been increasing as well, with 22.4% in 2012 (Center for Health Development, 2012) and 25.4% in 2016 (Center for Health Development, 2016).

Several studies have previously identified factors associated with CS delivery. In particular, older age (Amini, Mohammadi, Omani-Samani, Almasi-Hashiani, & Maroufizadeh, 2018; Amjad et al., 2018; Bayrampour & Heaman, 2010), high maternal BMI (overweight or obesity) (Al-Kubaisy, Al-Rubaey, Al-Naggar, Karim, & Mohd Noor, 2014; Amini et al., 2018; Chu et al., 2007; Gascho, Leandro, Ribeiro e Silva, & Silva, 2017), higher socioeconomic status (Amini et al., 2018; Amjad et al., 2018), unwanted pregnancy (Amini et al., 2018), lower parity (Amini et al., 2018; Gascho et al., 2017), pregnancy complications (Amjad et al., 2018), and larger infant head circumference (Amini et al., 2018) were determined as factors related to CS delivery.

Though it is well known that maternal obesity is a risk factor for CS (Al-Kubaisy et al., 2014; Amini et al., 2018; Chu et al., 2007), the impact of high maternal BMI on the risk of CS among pregnant women in Mongolia is still unclear. Elucidating whether high maternal BMI is related to CS delivery would be beneficial for policy makers and medical personnel to reduce the prevalence of obesity among women in Mongolia and for women in their efforts to reduce maternal weight.

Therefore, the aim of this study was to determine whether increased maternal BMI is associated with CS delivery in Mongolia.

## Methods

### Study Design and Population

This was a prospective observational study conducted with baseline and follow-up surveys in Darkhan-Uul Province, Mongolia. Participants were recruited from all 10 public health facilities, five health centers, three *Soum* (district) hospitals (all of them are primary health care facilities), and one general hospital, and one health agency in Darkhan-Uul Province, from November 2015 to January 2016.

Pregnant women who visited the above-mentioned health facilities for antenatal health checkups and presented with 20 weeks’ gestation or less were included in the study. Pregnant women who could not understand Mongolian or who found it psychologically challenging to participate in the study were excluded.

The participants were recruited by trained medical personnel, and baseline data were collected using self-administered questionnaires and body weight measurements.

A follow-up survey was conducted at Darkhan-Uul General Hospital. In Darkhan-Uul Province, the general hospital is the only health facility where women can give birth; therefore, all women would come to the hospital for delivery unless they move to another province. Data regarding information on mothers, infants, and mode of delivery were obtained from the hospital’s medical records.

### Baseline Survey

Sociodemographic data such as age, marital status, educational qualification, employment status, monthly household income, and the number of childbirths were collected using a questionnaire. Information regarding gestational age at the time of recruitment was also collected.

Maternal height was obtained from the participants, and if the participants did not know or were uncertain of their height, trained medical personnel performed the measurement. Participants’ body weight at recruitment was measured using a digital weighing scale (BC-758, TANITA Corp., Tokyo, Japan).

### Follow-up Survey

Information regarding childbirth, such as maternal pre-parturient weight, mode of delivery, gestational age at delivery, and the number of fetuses, was obtained from medical records at Darkhan-Uul General Hospital. Information on the estimated date of confinement (EDC) was also obtained from medical records to recalculate and confirm the gestational age at recruitment and delivery. Although information regarding infant’s birth weight and head circumference was also obtained, we decided to not include these data into analysis, because both these factors along with maternal BMI are predictors of cephalopelvic disproportion.

Data related to obstetric complications, such as gestational diabetes mellitus, preeclampsia, and fetal abnormality, were not available. Furthermore, information on the previous mode of delivery among multiparous women, fetal presentation, and placental presentation (placenta previa) could not be obtained.

### Recommended Weight Gain During Pregnancy

Since 1990, the Institute of Medicine (IOM) has recommended a range for gestational weight gain because overweight and obesity are risk factors for poor maternal and child health outcomes, and in 2009, the new guidelines presented recommended ranges for different categories of mothers during pre-pregnancy: BMI; 12.5–18.0 kg for underweight women, 11.5–16.0 kg for normal weight women, 7.0–11.5 kg for overweight women, and 5.0–9.0 kg for obese women (Institute of Medicine and National Research Council Committee to Reexamine IOM Pregnancy Weight Guidelines, 2009).

### Statistical Analysis

We compared the group of women who had vaginal deliveries with those who had CS deliveries using the chi-square test for categorical variables and Student’s *t*-test for continuous variables. Variables that correlated with CS delivery in bivariate analysis were selected as individual variables for logistic regression analysis. Before performing multiple logistic regression analysis, multicollinearity was evaluated using Spearman’s rank-correlation coefficient or Pearson’s product-moment correlation coefficient; if the coefficients exceeded 0.7, one of the variables was removed from multiple logistic regression analysis. We also performed bivariate and multivariate logistic regression analyses to calculate the odds ratios and *p* values.

All data were analyzed using IBM SPSS Statistics 25.0 for Windows (IBM Corp., Armonk, NY, USA). Two-tailed *p* values of <.05 were considered statistically significant.

### Ethics Considerations

This research project was approved by the Research Ethics Committee of the Graduate School of Medicine, The University of Tokyo, Japan (No. 10934), and the Ethical Review Board of the Ministry of Health, Mongolia (No. 06, November 19, 2015). All participants were clearly explained the aims of the study and the confidentiality of data. Written informed consent was obtained from all participants.

## Results

### Participants’ Characteristics

A total of 508 pregnant women agreed to participate in this study, of whom 15 participated in the baseline survey twice at different health facilities and thereby, duplicates were excluded from the study. Thus, data of 493 women were used for the baseline survey. After childbirth, data of 356 women were available from medical records, from which we obtained information on gestational age and EDC, and recalculated the gestational age at baseline survey for confirmation. Furthermore, we excluded 20 women as their gestational age at baseline survey exceeded 20 weeks. As a result, the data of 336 women were analyzed.

The study participants’ demographic characteristics are shown in Table 1. The maternal age (mean ± SD) was 28.1 ± 5.8 years; 273 (81.2%) women were married, 173 (51.5%) had graduated from university, and 140 (41.6%) were employed. The mean gestational age at recruitment was 12.9 ± 4.4 weeks, maternal BMI at the first antenatal care visit was 23.7 ± 4.0 kg/m^2^, maternal BMI at recruitment was 24.2 ± 3.9 kg/m^2^, and gestational weight gain was 12.0 ± 6.1 kg. Of the 336 women, 50 (14.9%) had CS delivery. The results of the bivariate analysis showed that maternal age at recruitment, employment status, maternal weight at the first antenatal care visit, maternal weight at recruitment, maternal BMI at recruitment, pre-parturient maternal weight, pre-parturient maternal BMI, gestational age at childbirth, and gestational weight gain, were significantly correlated with the mode of delivery. However, maternal weight at the first antenatal care visit, maternal BMI at the first antenatal care visit, maternal weight at recruitment, maternal BMI at recruitment, pre-parturient maternal weight, and pre-parturient maternal BMI significantly correlated with each other, and the correlation coefficient exceeded 0.7. Therefore, we decided to remove all variables from multiple logistic regression analysis, except for maternal BMI at the first antenatal care visit.

**Table 1:** Characteristics of Pregnant Women (n = 336)

	Total		Vaginal delivery		Cesarean section		*p*
	(*n* = 336)		(*n* = 286, 85.1%)		(*n* = 50, 14.9%)		
	Mean ± SD or *n* (%)		Mean ± SD or *n* (%)		Mean ± SD or *n* (%)		
Maternal age at recruitment (years)	28.1 ± 5.8		27.7 ± 5.6		30.6 ± 6.4		.001
Marital status							.119^a^
Single (divorced, widowed)	59	(17.6)	54	(91.5)	5	(8.5)	
Married	273	(81.2)	228	(83.5)	45	(16.5)	
Missing	4	(1.2)	4	(100.0)	0	(0.0)	
Educational status							.982^a^
≤Lower secondary school	33	(9.8)	28	(84.8)	5	(15.2)	
Upper secondary school	125	(37.2)	106	(84.8)	19	(15.2)	
≥University	173	(51.5)	148	(85.5)	25	(14.5)	
Missing	5	(1.5)	4	(80.0)	1	(20.0)	
Employment status							.016^a^
Employed	140	(41.6)	117	(83.6)	23	(16.4)	
Self-employed	51	(15.2)	48	(94.1)	3	(5.9)	
Nomad	12	(3.6)	7	(58.3)	5	(41.7)	
Unemployed	127	(37.8)	109	(85.8)	18	(14.2)	
Missing	6	(1.8)	5	(83.3)	1	(16.7)	
Monthly household income^b^							.679^a^
≤ 400,000	88	(26.2)	77	(87.5)	11	(12.5)	
410,000–800,000	165	(49.1)	138	(83.6)	27	(16.4)	
≥ 810,000	72	(21.4)	60	(83.3)	12	(16.7)	
Missing	11	(3.3)	11	(100.0)	0	(0.0)	
Childbirth experience							.492^a^
Primiparous	74	(22.0)	61	(82.4)	13	(17.6)	
Multiparous	231	(68.8)	198	(85.7)	33	(14.3)	
Missing	31	(9.2)	27	(87.1)	4	(12.9)	
Maternal height (cm)	160.0 ± 6.0		159.9 ± 5.8		160.1 ± 7.5		.897
Maternal weight at first antenatal care visit (kg)^c^	61.0 ± 11.4		60.3 ± 10.8		64.4 ± 13.8		.029
BMI at first antenatal care visit (kg/m^2^)^c^	23.7 ± 4.0		23.5 ± 3.9		25.0 ± 4.6		.022
<18.5	19	(5.7)	16	(84.2)	3	(15.8)	.276
18.5–25.0	165	(49.1)	144	(87.3)	21	(12.7)	
25.0–30.0	80	(23.8)	62	(77.5)	18	(22.5)	
≥30.0	20	(5.9)	17	(85.0)	3	(15.0)	
Missing	52	(15.5)	47	(90.4)	5	(9.6)	
Maternal weight at recruitment (kg)^d^	62.0 ± 10.9		61.4 ± 10.4		65.5 ± 12.9		.014
BMI at recruitment (kg/m^2^)^d^	24.2 ± 3.9		24.0 ± 3.8		25.5 ± 4.2		.012
18.5	14	(4.2)	14	(100)	0	(0.0)	.136
18.5–25.0	194	(57.7)	167	(86.1)	27	(13.9)	
25.0–30.0	96	(28.6)	81	(84.4)	15	(15.6)	
≥30.0	31	(9.2)	23	(74.2)	8	(25.8)	
Missing	1	(0.3)	1	(100.0)	0	(0.0)	
Gestational age at recruitment (weeks)	12.9 ± 4.4		12.9 ± 4.4		12.9 ± 4.2		.909
Pre-parturient maternal weight (kg)^e^	73.1 ± 11.9		72.2 ± 11.4		78.4 ± 13.5		.001
Pre-parturient BMI (kg/m^2^)^e^	28.5 ± 4.2		28.2 ± 4.1		30.5 ± 4.5		<.001
Gestational age at childbirth (weeks)^e^	38.7 ± 1.7		38.8 ± 1.6		37.9 ± 1.9		.001
Preterm (≤36 weeks)	29	(8.6)	22	(75.9)	7	(24.1)	.119
Term (>37 weeks)	304	(90.5)	263	(86.5)	41	(13.5)	
Missing	3	(0.9)	1	(33.3)	2	(66.7)	
Gestational weight gain (kg)^f^	12.0 ± 6.1		11.6 ± 6.0		13.9 ± 6.4		.023
Number of fetus							.161^g^
Single	331	(98.5)	283	(85.5)	48	(14.5)	
Multiple	5	(1.5)	3	(60.0)	2	(40.0)	

Student *t*-test. BMI: body mass index. Missing data were excluded from analysis.

^a^ Chi-squared test.

^b^ ₮20,000 . US$10.

^c^ Missing for 52 participants.

^d^ Missing for 1 participant.

^e^ Missing for 3 participants.

^f^ Missing for 55 participants.

^g^ Fisher’s exact test.

### Factors Related to CS Delivery

Table 2 shows the results of multiple logistic regression analysis for factors related to CS. A higher maternal BMI at the first antenatal care visit (adjusted odds ratio [AOR] = 1.102, 95% confidence interval [CI]: 1.008–1.205), higher gestational weight gain (AOR = 1.111, 95% CI: 1.041–1.185), older maternal age (AOR = 1.076, 95% CI: 1.007–1.150), and lower gestational age at childbirth (AOR = 0.765, 95% CI: 0.635–0.923) were significantly associated with CS delivery. Furthermore, compared to employed women, self-employed women had a lower risk of CS delivery (AOR = 0.241, 95% CI: 0.065–0.898).

**Table 2:** Factors Related to Cesarean Section Delivery

	Crude odds ratio	95% CI	*p* value	Adjusted odds ratio	95% CI	*p* value
BMI at first antenatal care visit	1.092	(1.012–1.178)	.024	1.102	(1.008–1.205)	.033
Gestational weight gain (kg)	1.065	(1.008–1.124)	.024	1.111	(1.041–1.185)	.001
Maternal age at recruitment (years)	1.090	(1.035–1.148)	.001	1.076	(1.007–1.150)	.030
Employment status						
Employed	Reference			Reference		
Self-employed	0.318	(0.091–1.109)	.072	0.241	(0.065–0.898)	.034
Nomad	3.634	(1.060–12.452)	.040	3.534	(0.648–19.278)	.145
Unemployed	0.840	(0.430–1.641)	.610	0.740	(0.326–1.676)	.470
Gestational age at childbirth (weeks)	0.781	(0.667–0.916)	.002	0.765	(0.635–0.923)	.005

Multiple logistic regression analysis adjusted for the variables in this table. CI: confidence interval; BMI: body mass index.

## Discussion

This study is the first to identify that high maternal BMI and gestational weight gain are associated with CS delivery in Mongolia. Furthermore, older maternal age and lower gestational age at childbirth were found to be associated with CS delivery.

### Participants’ Characteristics and Prevalence of CS

In this study, we analyzed the data of 336 women, although 508 women participated in the baseline survey. We could not follow-up with the remaining women because we excluded those who answered the questionnaires twice at baseline, and women whose gestational age at the baseline survey exceeded 20 weeks. Furthermore, some pregnant women may have had miscarriage while others may have delivered in other provinces.

Among the participants, only 50.7% had graduated from a university, though it is reported that 76.5% of women enroll to avail tertiary education in Mongolia (United Nations Educational, Scientific and Cultural Organization, 2018). Furthermore, it has been reported that the rate of CS delivery was 18.8% in Darkhan-Uul Province in 2016 (Center for Health Development, 2016); however, the rate was 14.9% in our study. Given these characteristics, we can imply that our participants might not represent the general population of pregnant women in Darkhan-Uul.

### Factors Related to CS Delivery

In this study, women with a higher maternal BMI had higher odds of CS delivery. This finding coincides with those of previous studies (Amini et al., 2018; Chu et al., 2007). Furthermore, women with higher gestational weight gain also had higher odds of CS delivery. In Mongolia, approximately half of the adult population are either overweight or obese (BMI ≥ 25.0 kg/m^2^) (Public Health Institute of the Ministry of Health and Sports, 2013), and obesity is a risk factor for several lifestyle-related diseases such as hypertension, cardiovascular diseases, and diabetes mellitus (Global Burden of Metabolic Risk Factors for Chronic Diseases Collaboration [BMI Mediated Effects] et al., 2014; Prospective Studies Collaboration et al., 2009). Additionally, obesity during pregnancy is a risk factor for miscarriage, preeclampsia, venous thromboembolism, and gestational diabetes mellitus (Fitzsimons et al., 2009); thus the importance of weight control should be discussed with women not only for reducing the risk of CS delivery but also for preventing lifestyle-related diseases in future. However, only 50 women had CS delivery in this study, of which 18 were overweight and 3 were obese; thus, we were unable to demonstrate a strong relationship between maternal BMI and CS delivery because of the small sample size. Nevertheless, information on nutrition such as intake of calories, micronutrients (i.e. several vitamins [Vitamin A, Vitamin B1, Vitamin B6, Vitamin B12, folic acid, Vitamin C and E, etc.] and minerals [iron, calcium, magnesium, etc.]) during pregnancy should also be provided to women.

Older women were more likely to have CS delivery in this study, which supports the finding of several previous studies (Amini et al., 2018; Amjad et al., 2018; Bayrampour & Heaman, 2010). However, the underlying reasons are not clear. One possibility is that older women may be more prone to pregnancy complications, resulting in CS (Amjad et al., 2018; Lin, Sheen, Tang, & Kao, 2004). In this study, we could not adjust for pregnancy complications such as preeclampsia, fetal distress, or history of CS because of a lack of information on these. Moreover, we could not obtain information on whether these were emergency CS cases or on the specific underlying reasons of CS delivery, such as abruptio placentae, failure to dilate, failure to descend, fetal distress, and meconium staining, which are not directly related to maternal BMI; adjustment for these variables should be considered in future research.

We also found that low gestational age at delivery was associated with CS, which is novel and previously unreported. A low gestational age at delivery being associated with CS may be due to the fact that women with pregnancy complications undergo CS before term, though we did not consider maternal pregnancy complications in our analysis. Further research is required to clarify the relationship between gestational age and CS delivery.

### Strengths and Limitations

This is the first study to examine whether high maternal BMI is associated with CS delivery in Mongolia. In recent years, being overweight or obese has become one of the major health issues, and the rate of CS has been increasing in Mongolia. Thus, identifying the relationship between high BMI and CS delivery would be useful for educating women of reproductive age on the importance of weight control.

Despite its strengths, the study has several limitations. First, the sample size was small in our study. The CS rate in our study (14.9%) was lower than that in the national report of 2016 (18.8%) (Center for Health Development, 2016). Moreover, 14.9% is not a very high rate for CS delivery; thus, caution must be exercised when interpreting the results of this study. Moreover, the results cannot be generalized to the entire Mongolian population. Second, we used women’s weight at the first antenatal care visit for analysis instead of women’s pre-pregnancy weight. In Mongolia, very few people have access to a weighing scale at home; therefore, it was difficult to obtain accurate information about the pre-pregnancy weight. Third, data regarding obstetric complications, such as gestational diabetes mellitus, preeclampsia, and fetal abnormality, were not available, and were thus, excluded in our analysis. Fourth, information on the previous mode of delivery among multiparous women, fetal presentation, and placental presentation (placenta previa) could not be obtained; thus, the underlying reasons for CS delivery could not be considered in this study.

## Conclusions

Our study determined that high maternal BMI and gestational weight gain were associated with CS delivery in the Darkhan-Uul Province, Mongolia. The increasing prevalence of overweight or obesity worldwide is one of the main health issues; thus, identifying whether high maternal BMI is associated with CS delivery would be beneficial not only for medical personnel but also for policy makers and women in their efforts to reduce weight and the prevalence of obesity in Mongolia. Further research covering all provinces in Mongolia should be conducted to validate our findings.

## Declaration of Conflicting Interests

The authors declared no potential conflicts of interest with respect to the research, authorship, and/or publication of this article.

## Funding

This study was conducted using funds from the Japan International Cooperation Agency (JICA) Partnership Program and Research Assistant Program from the Graduate School of Medicine at the University of Tokyo.
